# Effect of Humic Substances as Feed Additive on the Growth Performance, Antioxidant Status, and Health Condition of African Catfish (*Clarias gariepinus*, Burchell 1822)

**DOI:** 10.3390/ani11082266

**Published:** 2021-07-31

**Authors:** Markéta Prokešová, Milena Bušová, Mahyar Zare, Hung Quang Tran, Eliška Kučerová, Anna Pavlovna Ivanova, Tatyana Gebauer, Vlastimil Stejskal

**Affiliations:** 1 Faculty of Fisheries and Protection of Waters, South Bohemian Research Center of Aquaculture and Biodiversity of Hydrocenoses, Institute of Aquaculture and Protection of Waters, University of South Bohemia in České Budějovice, Husova tř. 458/102, 370 05 České Budějovice, Czech Republic; mzare@frov.jcu.cz (M.Z.); htranquang@frov.jcu.cz (H.Q.T.); aivanova@frov.jcu.cz (A.P.I.); tgebauer@frov.jcu.cz (T.G.); stejskal@frov.jcu.cz (V.S.); 2 First Faculty of Medicine, Institute of Hygiene and Epidemiology, Charles University and General University Hospital in Prague, Studničkova 7, 128 00 Prague, Czech Republic; milena.busova@lf1.cuni.cz (M.B.); el86kucerova@gmail.com (E.K.)

**Keywords:** biochemical parameters, Clariidae, fish nutrition, glutathione, Siberian leonardite

## Abstract

**Simple Summary:**

Global aquaculture requires the development of new strategies to maintain the continuous growth of production, such as the development of aquafeeds with sustainable and functional components, ensuring better growth and health conditions of fish. Humic substances (HS) have the potential to become a functional additive for aquafeeds, as their growth-promoting and immunostimulant effects have been found in farm animals. Recently, there is limited knowledge on how different HS affects overall performance of various fish species. Therefore, in this study, the effects of four experimental Siberian leonardite HS diets (HS0, HS1, HS3, and HS6) on growth and production parameters, condition and somatic indices, overall mortality, health condition, and antioxidant status were assessed in juvenile *Clarias gariepinus,* which is well-known as a fast-growing and high-resistant fish species when reared at high stocking densities up to 500 kg m^−3^. In this study, growth and production parameters, condition, and somatic indices or mortality rate were not significantly affected by tested HS diets. On the other hand, moderately positive effects were observed regarding health status and good antioxidant parameters, especially in the HS3 group over the 56-day study.

**Abstract:**

In the present study, a possible nature immunostimulant and growth promoter—humic substances (HS) originating from Siberian leonardite mineraloid—were tested on juvenile *Clarias gariepinus* performance. Feed additive was applied onto commercial pelleted feed at four HS levels—0, 1, 3, and 6% w/w (HS0, HS1, HS3, HS6, respectively). Diets were tested in five repetitions (in total, *n* = 1800 individuals, mean body weight 28.1 ± 6.2 g) for 56 days. Growth and production parameters, fish condition and somatic indices, and overall mortality were evaluated after 14, 28, 42, and 56 days of exposure. Whereas, plasma samples were collected only after 0, 28, and 56 days, when fish health status was assessed with biochemical parameters (total proteins, TP; alanine aminotransferase, ALT; aspartate aminotransferase, AST; lactate dehydrogenase, LDH; cholesterol, CHOL; triglycerides, TAG) and fish antioxidant status with glutathione (reduced glutathione, GSH; oxidized glutathione, GSSG; glutathione ratio GSH/GSSG). Although a significantly positive effect of HS feed additive on growth performance was not found in the present study, moderately positive effects were found regarding biochemical parameters (ALT, AST, LDH, CHOL, and TAG) and antioxidants (GSH/GSSG ratio) that were improved especially in the HS3 group.

## 1. Introduction

Global fish consumption is predicted to continuously grow together with the world population. While the global trend is to reverse capture fisheries due to overfishing, the aquaculture sector has the potential to continuously increase. Further growth of aquaculture demands new sustainable development strategies [1]. For instance, improving aquafeeds using new sustainable alternative components—insect and plant meal, microbial biomass, food waste [2], and functional additives—organic acids, medicinal herbs, probiotics etc. [3] can contribute to increasing fish production through improved feed utilization, growth performance, overall health condition, disease resistance, and survival rate of fish, and therefore lower economical losses to fish farmers.

African catfish, *Clarias gariepinus* (Burchel, 1822), is a promising freshwater fish species suitable for intensive aquaculture due to its fast growth (SGR 2.28% day^−1^) at high stocking densities (up to 500 kg m^−3^) [4], excellent food conversion (FCR 0.87–0.97) [5], an ability to breathe atmospheric air due to the accessory suprabranchial respiratory organ, a tolerance to extreme environmental and poor water quality conditions—dissolved oxygen < 4.0 mg L^−1^ [6], ammonia concentration < 0.34 mg L^−1^ [7], presence of substances associated with water re-use [5], as well as tasty flash of high nutritive value [8,9]. Therefore, the annual aquaculture production of *C. gariepinus* grew from 5308 to 240,860 tons since the year 2000 [10,11].

Humic substances (HS) have the potential to be used as functional additives in aquafeeds. They represent a class of ubiquitous compounds resulting from microbial decomposition of organic matter, mainly from plants, and, at present, the most common form of organic carbon that naturally occurs in fresh waters [12,13,14]. Generally, at least three forms of HS—humin, fulvic, and humic acids (abbreviations HU, FA, and HA, respectively)—are distinguished based on their water solubility, molecular weight, and oxygen and nitrogen content [12,15,16]. The major elements in composition are C, O, H, N, and S; they are always present regardless of their origin [16]. The presence of functional groups (such as carboxylic, phenolic, ketonic, aromatic, aliphatic, etc.) enable HS to interact with both living and non-living matter [17]. Their chemical structure is very diverse and different HS can therefore provoke contrasting effects [18]. Humic substances are applied in agriculture, environmental aspects, or biomedicine due to their specific antifungal, antiseptic, antioxidant, and detoxifying properties [16,19].

Recent research has confirmed that HS can be taken up and bioconcentrated by aquatic organisms (macrophytes, invertebrates, and vertebrates) after short-term exposure [14,20]. Recently, few studies tested the effects of HS in fish species. For instance, HS exposure (diet or water application) was found to have beneficial effects on growth performance of *Cyprinus carpio* [21,22], *Oncorhynchus mykiss* [23], *Paramisgurnus dabryanus* [24], and *Xiphophorus helleri* [25]. Furthermore, the HS-enriched diet or water were found to improve microflora community diversity of *P. dabryanus* [24] and *O. mykiss* [26], lysozyme antibacterial activity of *C. carpio* [21] and *P. dabryanus* [24], recovery after stressful handling in *X. helleri* [25], and resistance against bacterial (*Aeromonas hydrophila*, *Aeromonas salmonicida, Yersinia ruckeri*) infection in *C. carpio* [21,27] and *O. mykiss* [26] or parasitic infection (monogenea *Gyrodactylus turnbulli* and *Dactylogyrus* sp.) in *Poecilia reticulata* [28]. On the other hand, undesirable effects of HS exposure were also observed, for example, on the growth of *Rhamdia quelen* [29]; gill, liver, and kidney histology of *C. carpio* [22]; or sex ratio in *X. helleri* [25]. However, effects of dietary HS application have not yet been studied in juvenile *C. gariepinus.*

The aim of the present study was to broaden knowledge on how HS feed additive (0, 1, 3, and 6% inclusion level) originating from Siberian leonardite mineraloid affects the growth, antioxidant status, and health performance of juvenile *C. gariepinus* during a 56-day exposure.

## 2. Materials and Methods 

### 2.1. Experimental Design

Juvenile *C. gariepinus* was imported from AGRO Fish Farm Ltd. (Handlová, Slovakia) to the Institute of Aquaculture and Protection of Waters (České Budějovice, Czech Republic). 

After transport, 100 fish (mean weight 28.1 ± 6.2 g) were placed into each of 20 rearing tanks and acclimated for one week. At the start of the experiment, 90 fish (mean body weight 32.4 ± 2.0 g and total body length 16.2 ± 3.4 cm) were left in each tank (stocking density 49.0 ± 1.5 kg m^−3^). In total, four HS diets were tested in five repetitions (*n =* 5 tanks/group):

HS0—control diet without HS supplement (0 g/100 g of feed);
HS1—diet with 1% of HS (1 g/100 g of feed);
HS3—diet with 3% of HS (3 g/100 g of feed); and
HS6—diet with 6% of HS additive (6 g/100 g of feed).

Each diet was prepared from commercial pelleted food (2 mm Aller Performa and 3 mm Bona Float, Aller Aqua, Golub-Dobrzyń, Poland) and spray-enriched with commercial HS feed additive HUMAFIT (HUMÁTY s. r. o., Sadov, Czech Republic) containing about 5% of dry matter, the Siberian leonardite mineraloid at a 68% HS content with FA:HA ratio 94.8:5.2, non-toxic elemental composition (Table 1), and carbon content of 39.1% (total organic—38.9% and inorganic carbon—0.2%). Thereafter, food was dried in a laboratory dryer (UN 75, Memmert GmbH + Co. KG, Schwabach, Germany) at 40 °C for 24 h; a similar procedure was used by [27,30]. Table 2 shows proximate composition of the experimental diets.

The four groups were reared in four recirculation aquaculture systems (RAS) with tap water. Each RAS consisted of a submerged biofilter tank (volume 300 L) with bio-elements (BT 10, Ratz Aqua & Polymer Technik GmbH, Remscheid, Germany), five rectangular 60 L rearing tanks, and a 300 L sump tank with filter foam (Bioakvacit PPI 10, Jezírka Banat, s. r. o., Hněvotín, Czech Republic). Water flow rate 3.6 L min^−1^ was maintained in rearing tanks using a regulated pump (Jecod-Jebao DCS 12000, Jecod Co., Ltd., Dongsheng, China). Starter bacteria encouraged biofilter function (Bakterienstarter, Tripond, Möhnesee, Germany) to support growth of nitrifying bacteria [31]. Levels of NH_3_, NO_2_^-^, and NO_3_^-^ were checked weekly by an automatic method of detection using LCK cuvette tests with barcode and spectrophotometer (DR 2800, Hach Lange, Prague, Czech Republic). Water was heated to 27 ± 0.5 °C using eight aquarium heaters (Jäger 3619, Eheim GmbH & Co. KG, Deizisau, Germany). Oxygen concentration of outlet water was maintained at 60 ± 12% (air pump Airmac DBMX80, Air Mac, Inc., Dallas, Texas) and the pH value was about 7 ± 0.3. Water temperature, pH, and oxygen levels were measured twice a day using a multimeter (HQ40d multi, Hach Lange s. r. o., Prague, Czech Republic). The light regime was 12 L:12 D with the light period 08.00–20.00 h. The light intensity above the water surface was set up to 500 lx (light meter DT-8809, Cem GmbH, Kamp-Lintfort, Germany).

The daily food ration was adjusted according to manufacturer’s instructions (initially 4%, gradually reduced to 2% biomass weight). Fish in each tank were fed using two automatic feeders (Twin, Eheim GmbH & Co. KG). The daily food ration was served to fish 12 times a day to prevent cannibalism, and thus the mortality incidences [32]. The fish ate all of the food almost immediately after feeding and, on the rare occasion that they did not, the remaining floating pellets were caught from rearing tanks with a net 30 min after feeding. The dry weight of uneaten pelleted food and mortality cases were recorded each day, if present. Furthermore, excrements were cleaned daily from the tanks through sedimentation cones. The drained water (~1/3 of each rearing tank volume) was replaced with tap water. The trial lasted for 56 days.

### 2.2. Sampling and Calculations 

Every 14 days (days 0, 14, 28, 42, and 56), fish were starved for one day. On the next day, the weight of biomass (B, g) and the count of all fish inside each tank was recorded. Thereafter, randomly selected fish (*n* = 20 fish/tank, all fish/tank only selected on day 56) were anesthetized in clove oil (0.03 mL/L^−1^ of water), individually weighed (W, g), and measured for total (TL, mm) and standard body length (SL, mm). By the end of the experiment, 10 fish/group were sacrificed (blown to the head) and dissected for collection of liver and spleen that were then weighed (accuracy 0.0001 g). Based on the recorded data, selected growth and production parameters, condition and somatic indices, and survival rate were calculated using the following equations:

Survival rate, SR (%) = (N_f_ × 100)/(N_i_–N_s_)

Specific growth rate, SGR (% day^−1^) = [(lnW_f_ − lnW_i_)/t] × 100

Absolute growth rate, AGR (g day^−1^) = (W_f_ −W_i_)/t

Food conversion ratio, FCR = F/(B_f_ − B_i_)

Daily feeding rate, DFR (feed fish^−1^ day^−1^) = (TDFI/N_f_/t)

Coefficient of variation, CV (%) = (SD/W_m_)

Condition factor, K = (W_f_/TL^3^) × 100

$$\mathrm{Thermal}\;\mathrm{growth}\;\mathrm{coefficient},\;\mathrm{TGC}=[(∛W_{f}-∛W_{i})/(T\;\times \;t)]\;\times \;1000$$

Hepato-somatic index, HSI (%) = W_liver_ × 100/BW

Splenic-somatic index, SSI (%) = W_spleen_ × 100/BW,

where N_f_ = final number of fish per tank, N_i_ = initial number of fish per tank, N_s_ = number of sampled fish per tank, W_f_ = final mean individual weight (g), W_i_ = initial mean individual weight (g), t = fattening period (days), T = temperature (°C), SD = standard deviation of weight, W_m_ = mean individual weight (g), F = total feed consumption (g), B_f_ = final fish biomass (g), B_i_ = initial fish biomass (g), BW = body weight of fish (g), TDFI = total dry feed intake (g), TL and SL = total and standard body length (cm), W_liver_ = liver weight (g), and W_spleen_ = spleen weight (g).

### 2.3. Analysis of Blood Parameters 

#### 2.3.1. Biochemical Parameters 

Blood samples were taken from two fish per tank (*n* = 10 fish/group) on days 0, 28, and 56. Prior to the sampling, fish were anesthetized in a clove oil bath (0.03 mL L^−1^ water) and starved for one day. Blood was collected using heparinized syringe (50 IU μL^−1^) from the caudal vessels. After the sampling, fish were recovered in clean aerated water and returned to the tank [33]. Blood samples for analysis of biochemical parameters were collected in plastic tubes (Vacuette) with EDTA (ethylenediaminetetraacetic acid) as an anticoagulant for blood plasma preparation after separation (at 4 °C, 10 min at 3000 rpm, centrifuge Mikro 200R, Hettich, Beverly, Massachusetts). Separated plasma was used for biochemical analysis (Stafila laboratory, České Budějovice, Czech Republic) of total proteins (TP), alanine aminotransferase (ALT), aspartate aminotransferase (AST), lactate dehydrogenase (LDH), cholesterol (CHOL), and triglycerides (TAG). Simultaneously, part of a blood sample was pipetted directly into the plastic tube (Vacuette) with sodium fluoride (NaF) as an inhibitor of glycolysis. These samples were used for glucose (GLU) analysis.

#### 2.3.2. Antioxidant Status—Reduced (GSH) and Oxidized (GSSG) Glutathione

Immediately after blood collection, 100 μL of each whole blood sample for GSSG determination was transferred into 1.5 μL (Eppendorf) microtubes with scavenger, and 10 μL of M2VP (1-methyl-2-vinyl-pyridium-trifluoromethane sulfonate) to prevent oxidation of GSH to GSSG during sample preparation. Next, 50 μL of whole blood sample for GSH determination was transferred into 1.5 μL pure microtube (Eppendorf) without treatment. All samples were frozen at −80 °C until the analysis was carried out.

The results of reduced glutathione (GSH) and oxidized glutathione (GSSG), as markers of the antioxidant status of organism, were measured and calculated using an assay kit (GSH/GSSG-412, Bioxytech) by Ellman’s method (1959), which was modified by Tietze (1969). Immediately before analysis, samples were thawed and mixed. After all procedures and reactions with Ellman’s reagent (5,5′-dithiobis-2-nitrobenzoic acid, DTNB), the samples were measured by a spectrophotometric reader at 412 nm (Epoch, BioTek, Winooski, Vermont) in triplicates. The calibration curve was constructed using the standards of GSH (0.0, 0.1, 0.25, 0.5, 1.5, and 3.0 µM GSH), then GSH and GSSG concentrations of each sample were calculated. The antioxidant status of the fish organism was assessed by a GSH/GSSG ratio.

### 2.4. Statistical Analysis

Data are expressed as mean ± SD. Tanks were considered as the experimental units [34] for assessment of BW, B, TL, SL, SGR, AGR, FCR, DFR, CV, K, TGC, overall mortality, and somatic indices at selected time-points (0, 14, 28, 42, and 56 days). This is unlike biochemical parameters (TP, ALT, AST, CHOL, TAG, LDH, and GLU), antioxidant parameters (GSH, GSSG, and GSH/GSSG ratio), and somatic indices (HSI and SSI), when each fish was considered the experimental unit. Statistical analysis was performed using statistical software (STATISTICA 12.0, StatSoft). All data was tested for normal distribution by the Shapiro–Wilk test and homogeneity of variance was tested by the Hartley–Bartlett test. When these two conditions were met, ANOVA analysis was used for subsequent evaluation of data. Otherwise, a non-parametric Kruskal–Wallis test was used. The effects of HS diet, time, and their additive effects on selected parameters were tested by two-way ANOVA. After these analysis, Tukey’s HSD test was used for assessing significant differences among tested groups. The level of significance was considered at the 0.05 *p-*level. Moreover, correlation analysis of parameters mentioned above was created in R environment using statistical software RStudio Version 1.4.1103 (RStudio. Ink, Boston, Massachusetts). The relationship between two variables was considered based on the *r* value as: strong (*r* > 0.7), moderate (*r* 0.5–0.7), weak (*r* 0.3–0.5), very weak, or no correlation (*r* ˂ 0.3).

## 3. Results

In the present study, HS feed inclusion (HS0, HS1, HS3, HS6) had no significant effect (*p* > 0.05, one-way ANOVA) on initial BW, TL, SL, K, CV, SGR, FCR, and overall mortality of *C. gariepinus* juveniles after 14, 28, 42, and 56-day HS feeding exposure (Table 3 and Supplement S1–S4). Furthermore, somatic indices, HSI and SSI, showed no significant differences (*p* > 0.05) among groups after the 56-day HS exposure (Table 3). Additionally, B, AGR, DFR, and TGC did not differ significantly (*p* > 0.05) at the end of the experiment.

Two-way ANOVA analysis confirmed a significant increase (*p* < 0.05) of growth parameters (BW, TL, SL), K, CV, and overall mortality in time. On the other hand, it revealed a significant decrease (*p* < 0.05) of SGR, and no difference of FCR (*p* > 0.05) over time. Interaction effects of time and HS exposure were not significant (*p* > 0.05), and neither HS groups had no significant effect (*p* > 0.05) on selected parameters (BW, TL SL, SGR, K, CV, FCR, and overall mortality) over time.

Biochemical parameters (TP, ALT, AST, LDH, CHOL, and TAG) were not significantly different (*p* > 0.05) between the tested HS groups after 28- and 56-day exposure (Table 4 and Supplement S5). On the other hand, GLU differed significantly (*p* < 0.05) between groups over the experiment. After 28 days, GLU was significantly lowered in HS groups compared to the control group. Meanwhile, after 56 days, only GLU in the HS6 group was significantly (*p* < 0.05) lowered compared to other groups.

Glutathione results showed differed after 28- and 56-day exposure (Table 4 and Supplement S5). After 28 days, reduced glutathione (GSH) was not significantly different (*p* > 0.05) between groups, while oxidized glutathione (GSSG) and glutathione ratio (GSH/GSSG) significantly differed (*p* < 0.05) between groups. Oxidized glutathione was significantly (*p* < 0.05) the highest in the HS1 group, while it did not differ (*p* > 0.05) between other groups (HS0, HS3, HS6) with the lowest GSSG value in the HS3 group. The glutathione ratio was significantly (*p* < 0.05) the highest in the HS3 group, the lowest (*p* < 0.05) in the HS1 group, while it was not significantly (*p* > 0.05) different from other groups in HS0 and HS6. After 56 days, glutathione GSH, GSSG, and GSH/GSSG were not significantly different (*p* > 0.05) between groups, although the lowest GSSG and highest GSH/GSSG ratio were observed still in the HS3 group compared to other HS-rich groups. 

Two-way ANOVA analysis revealed no significant differences by HS groups or interaction of HS groups and time (*p* > 0.05) on tested biochemical parameters. However, significant changes (*p* < 0.05) were observed over time, when decreased (*p* < 0.05) ALT, AST, LDH, CHOL, and TAG levels were observed throughout the experiment. While GLU did not significantly differ by time (*p* > 0.05) over the experiment, TP significantly increased (*p* < 0.05) after 28-day exposure and declined (*p* < 0.05) after 56 days.

Two-way ANOVA analysis did not reveal significant interaction effects of HS groups with time of exposure (*p* > 0.05) or effect of HS groups alone (*p* > 0.05) on glutathione GSH, GSSG, and GSH/GSSG levels. Significant differences were observed over time (*p* < 0.05). While GSH and GSSG significantly decreased (*p* < 0.05) over time, the GSH/GSSG ratio showed a positive corelation with time (*p* < 0.05) over the experiment.

**Figure 1:**
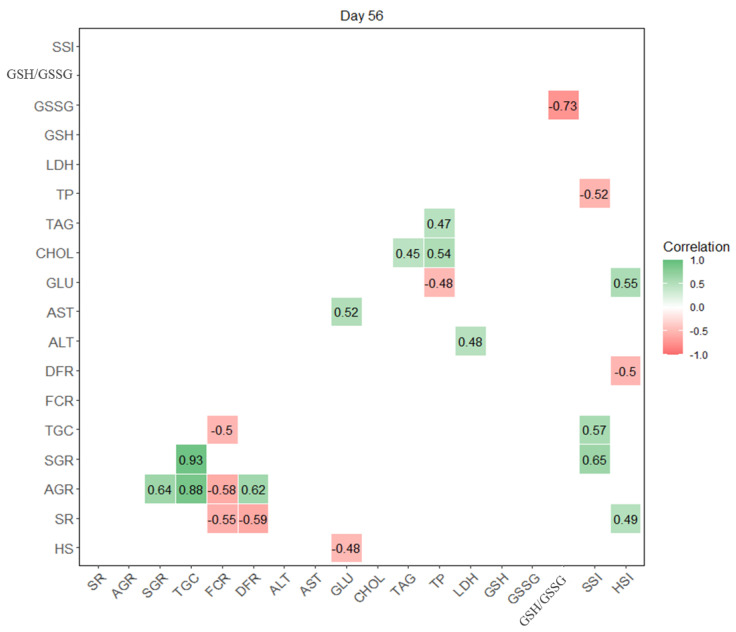
Correlation matrix between HS diets (0, 1, 3, 6%), growth (AGR, SGR, TGC), production (SR, FCR, DFR), biochemical (ALT, AST, GLU, CHOL, TAG, TP, LDH) and antioxidant (GSH, GSSG, GSH/GSSG) parameters and somatic indices (SSI, HSI) after 56 days of feeding. Negative correlations are coloured in red, and positive are in green, with a scale from –1 to +1 with 0 representing the absence of correlation (no colour in figure). Correlations are assessed as strong (positive > 0.7, negative ˂ −0.7), moderate (±0.5 to ±0.7), weak (±0.3 to ±0.5), and very weak (0 to ±0.3).

Because of the complex and potentially interactive effects between the abovementioned parameters after a 56-day HS exposure, the interrelationships were analysed using a correlation matrix (Figure 1). Strong positive correlations (*r* > +0.7) were found between TGC and AGR, and TGC and SGR, while a strong negative correlation (*r* ˂ −0.7) was found between the GSH/GSSG ratio and GSSG. Then, moderate (*r* from ±0.5 to ±0.7) positive correlations were observed between SGR and AGR, DFR and AGR, GLU and AST, TP and CHOL, SSI and SGR, SSI and TGC, and HSI and GLU. However, moderate negative correlations were found between FCR and SR, FCR and AGR, FCR and TGC, DFR and SR, GSH/GSSG and GSSG, SSI and TP, and HSI and DFR. Other tested correlations were weak (*r* from ±0.3 to ±0.5), very weak, or none (*r* from 0 to ±0.3).

## 4. Discussion

### 4.1. Humic Substances 

Diet or drinking water supplemented with HS was found to have beneficial effects on crop and livestock production [12,35,36]. Recently, positive effects of HS application have been found in fish, such as improved growth, intestinal microflora, lysozyme activity, stress, and disease resistance [23,24,27]. Nevertheless, there is still relatively low knowledge on how different HS sources and products (natural, synthetical, and commercial preparations) affect the performance of different fish species. In the present study, the effect of commercial HS feed additive HUMAFIT (HUMÁTY s. r. o., Czech Republic) containing about 5% of dry matter, the Siberian leonardite mineraloid (HS content 68% with FA:HA ratio 94.8:5.2, non-toxic elemental composition with C—39.1%, H—2.3%, N—0.4%, S—0.5%) was firstly assessed on growth, and antioxidant status and health performance of juvenile *C. gariepinus*. Unlike previous studies that compared their results with farm or laboratory animals, our study only compares with data originating from fish research.

### 4.2. Leonardite Mineraloid 

In general, leonardite is a dark coloured vitreous mineraloid which is a main source of HS (up to 90%). It was formed by natural lignite oxidation, therefore its HS chemical structure may differ in elemental composition [37,38,39]. The major elements in HS composition are C, O, H, N (45–60, 25–45, 4–7, 2–5%, respectively) and are always present regardless of their origin [16]. Toxicity of naturally occurring HS is remarkably low [40] and their effective concentrations for aquatic species possibly lie in an environmentally realistic range up to 100 mg C/L [13,41]. 

### 4.3. Growth Performance

In the present study, the HS-incorporated diet (HS0–6) had no significant effect on the growth performance of *C. gariepinus*, although the fish increased five-times their initial weight (BW, from 36.4 ± 2.0 g to 173.5 ± 8.0 g) during the 56-day experiment. Similarly, dietary humic acid sodium salt (0.3, 0.6, and 1.2%) had no significant effects on growth performance (BW, WG, SGR) of juvenile *Oncorhynchus mykiss* after 60 days [26]. On the other hand, the next studies reported enhanced growth after use of HS feed additive. For instance, HA (0.4, 0.8, 1%) and lignite FA (0.5, 1.0, 1.5, 2%) feed inclusion had a positive effect on *Cyprinus carpio* and *Paramisgurnus dabryanus* growth after 45- and 60-day exposure, respectively [21,24].

Moreover, it was found that HS-enriched water may influence fish growth. For example, although [29] no impact of HA-enriched water (10, 25, and 50 mg/L) on *Rhamdia quelen* growth after a 40-day exposure was observed, Lieke et al. and Meinelt et al. [23,25] reported that the use of water with commercial FA (10 and 50 mg C/L) and synthetic HS (5, 30, and 180 mg C/L) provided a positive effect on *O. mykiss* and *Xiphophorus helleri* growth after 28-day and 21-week exposure, respectively. 

Based on previous research, the HS-enriched aquafeed (0.3–2%) and water (5–180 mg C/L) may have positive/none/negative effects on fish growth. Therefore, further research is required. To the HS application method, Lieke et al. [23] adds that “unlike a bath, the feed/oral application method may have several downsides, because of feed competition, fish ingest different amounts of feed, thus different amounts of additive, which makes the effects inaccurate” (explaining that too low concentrations can have no effects, while too high may exert adverse effects). On the other hand, for example, Yamin et al. [27] reported no conclusions regarding the type of HS application method (water, feed, or a combination of both) on the growth and infection rate of *C. carpio*, although he tested three different HS sources (leonardite mineraloid, commercial synthetic HA, and HS-rich water from *Oreochromis* culture RAS). 

### 4.4. Food Conversion Ratio

Food conversion ratio describes the weight gain from a given quantity of consumed feed. In our study, FCR was not significantly influenced between HS groups. Mean FCR was 1.07 ± 0.14 during the experiment. This matched the FCR range 0.82–1.17 reported for juvenile *C. gariepinus* [42,43].

In accordance with our study, Yilmaz et al. [26] did not observe any significant improvement in FCR and growth of *O. mykiss* after 60 days of feeding with 0.3–1.2% HA sodium salt. Unlike our study, Lieke et al. [23] reported reduction in FCR in juvenile *O. mykiss* after 28-day exposure to FA-rich water, significantly in 50 mg C/L. Furthermore, Gao et al. [24] reported promoted FCR and growth (W, WG, and SGR) in juvenile *P. dabryanus* fed lignite FA (0.5–2%) for 60 days, with the best results after use of 1.5% diet. 

Gao et al. [24] explained that *P. dabryanus* improved FCR and that growth could be associated with observed increased digestive enzyme (protease, lipase) activities after 60-day lignite FA exposure in their study. Moreover, Yilmaz et al. [26] recorded increased digestive enzyme (stomach pepsin, intestinal amylase, trypsin, and lipase) activities with dietary HA sodium salt (notably with 0.6% inclusion), although growth of *O. mykiss* was not improved. Furthermore, Gao et al. [24] observed increased and more diversified intestinal microflora of *P. dabryanus* (reduction in *Serratia*, *Acinetobacter*, *Aeromonas,* and *Edwardsiella*, while enhancement of *Lactobacillus* in relative abundance). Based on these data, some HS sources orally applied can have beneficial effects on fish growth, feed utilization, and intestinal health condition. 

### 4.5. Condition Factor and Somatic Indices 

In the present study, condition factor K (the length/weight relationship) and somatic indices HSI and SSI were assessed as indicators of fish health. However, they showed no significant differences between HS groups after 56-day exposure. This means that fish were at similar body conditions in all HS groups by the end of our experiment. 

In accordance with our study, Lieke et al. [23]—who tested low (5 mg C/L) and high (50 mg C/L) concentrations of FA-rich water—reported no effect on juvenile *O. mykiss* condition factor K, HSI, SSI, as well as fat content after 28-day exposure. On the contrary, Abdel-Wahab et al. [21] reported improved *C. carpio* condition factor K in commercial HA fed groups (0.4–1%). Furthermore, Yilmaz et al. [26]—who fed *O. mykiss* a HA sodium salt diet (0.3–1.2%)—found significantly higher HSI in the 1.2% group compared to others after 60-day exposure, which might be a negative effect related to stress and body detoxification processes. Nevertheless, the lower supplemented groups 0.3 and 0.6% did not have a negative impact on *O. mykiss* HSI. Besides, they found a significantly lower viscerosomatic index (VSI) in *O. mykiss* fed by all HA sodium salt diets (0.3–1.2%), which may be attributed to a negative correlation between HA-enriched diets and fat level or weight of other visceral organs [26].

### 4.6. Biochemical Parameters

In the present study, selected plasma biochemical parameters (TP, ALT, AST, LDH, CHOL, TAG, and GLU) were assessed as possible determinants of the health and physiological condition of fish. Fish metabolism reflects the state of the living environment and is affected by changes in water chemistry and reproductive activity, nutritional status, etc. Therefore, fish are often used as acceptable bioindicators of environmental pollution [44]. In our study, constant, defined, and monitored conditions in the recirculation system were set for all tested groups (HS0-HS6), and the experimental fish were the same age with the same access to feed.

Total protein levels are mainly considered as a marker of kidney function and the immune state of an organism [45]. In our study, TP did not differ between tested groups (HS0–HS6) in *C. gariepinus* after 28 and 56 days of feeding exposure. Similarly, Yilmaz et al. [26] and Lieke et al. [23] also reported no differences of TP in *O. mykiss* after use of a HA sodium salt-supplemented diet (0, 0.3, 0.6, 1.2%) for 60 days or FA bath exposure for 28 days, respectively. By the end of our study, TP levels ranged between 26.9–32.3 g L^−1^, which is close to Abalaka’s [46] finding of TP content 32.09 ± 1.79 g L^−1^ for farmed *C. gariepinus*.

Enzymes ALT, AST, and LDH are related primarily to liver function; their increased levels may refer to liver dysfunction [45]. In this study, activity of ALT, AST, and LDH enzymes was within the species-specific optimal physiological range, and no significant differences were observed between the tested groups after 28 and 56 days of HS feeding. Our results are comparable with levels of AST 2.59 ukat L^−1^ and ALT 0.82 ukat L^−1^ observed in farmed *C. gariepinus* [46]. In our study, LDH levels did not significantly differ, as well as in Yilmaz et al.’s study [26] where they measured serum LDH of *O. mykiss* after a 60-day dietary HA sodium salt exposure. 

Plasma CHOL and TAG indicate the nutritional status of fish, and their fluctuations signalize disorders of lipid metabolism [45]. Our results confirmed no changes in *C. gariepinus* lipid metabolism exposed to HS-rich feed, as the plasma CHOL and TAG levels were not different between groups after 28 and 56 days of HS feeding exposure. By the end of the experiment, CHOL and TAG levels ranged between 2.68–2.93 mmol L^−1^ and TAG levels from 0.91–1.71 mmol L^−1^. In accordance with our study, Yilmaz et al. [26] recorded no change in the *O. mykiss* CHOL level, but they observed decreased TAG with increasing dietary HA sodium salt inclusion after 60 days. Our results also indicated a slight decrease in TAG levels in groups HS1 and HS6 compared to the control group HS0, while it was moderately increased in HS3 group after 28 and 56.

Glucose is a stress marker and its metabolism can reflect fish nutritional state. In the present study, GLU levels were significantly lower (3.88 ± 1.21 mmol L^−1^) in HS groups compared to the control group (6.21 ± 3.01 mmol L^−1^) after 28 days. Later (after 56 days), GLU levels were significantly lower (3.20 ± 1.37 mmol L^−1^) only in the HS6 group compared to the others. These GLU results reflected probably better fish resistance to stress in the HS6 group. 

Furthermore, a significant decrease in ALT, AST, LDH, CHOL, and TAG levels was recorded throughout the experiment. Therefore, it seems that HS addition had an evident beneficial effect that resulted in moderately reduced activity of ALT, AST, LDH, CHOL, and TAG in all tested HS groups (HS1, HS3, HS6). 

Biochemical markers in fish do not have set exact limits, so ranges of enzymes and other markers are therefore determined. It depends on the conditions in which the fish live (wild or farmed fish), as well as water chemistry, age, sex, feeding method and quality, nutritional status, or climatic conditions [45]. Therefore, the evaluation of these parameters should be considered in this context and the comparison with the results of other studies is indicative.

### 4.7. Antioxidant Status, Reduced (GSH) and Oxidized (GSSG) Glutathione 

In general, the antioxidant status of organisms can be monitored due to the measurability of the glutathione levels [47]. It is responsible for protection not only against ROS and reactive nitrogen species (RNS), but also for detoxification of an organism [48,49]. The oxidative stress and xenobiotics present in aquatic environment may suppress GSH levels due to the impairment of adaptive mechanisms in fish [50,51,52]. For instance, a decrease in GSH levels can be associated with certain pathologies in both humans and animals [49], while the ratio of glutathione (GSH/GSSG) may characterize the redox and health status of organisms [48]. Primarily, a higher GSH/GSSG ratio indicates a good status of organisms and their protection against potential damage [48]. 

Only a few results have been published about dietary HS and their effects on antioxidant fish health status. According to our results, the differences in the content of GSH, GSSG, and GSH/GSSG ratio between groups fed with the HS diet were statistically insignificant after 56 days of HS exposure. However, the GSH/GSSG ratio was the highest in the HS3 group compared to the other tested groups (HS0, HS1, HS6). After 28 days of HS exposure, the GSH/GSSG was 30% higher in the HS3 group than in the control group HS0. After 56 days of HS exposure, the GSH/GSSG ratio was 20% higher than the control group HS0, although the differences were not significant. These results proved moderate positive effect of the HS3 group and are in agreement with the lowest mean overall mortality observed in the HS3 group after 28, 42, and 56 days of dietary HS exposure. It seems that both the antioxidant glutathione status expressed as the GSH/GSSG ratio and the health condition of fish can be moderately improved by dietary HS additive in intensively reared *C. gariepinus*. In agreement with these results, the optimal levels of biochemical parameters were all in tested groups without abnormalities.

It can be concluded that the antioxidant defence system and health status of fish may be supported by dietary HS additives in intensively reared *C. gariepinus*, as the optimal, highest ratio GSH/GSSG was observed in fish with the HS3 diet. 

### 4.8. Overall Mortality

In the present study, a significantly positive effect of HS feed inclusion (0, 1, 3, 6%) on survival rate was not confirmed in juvenile *C. gariepinus*. Although the overall mortality did not differ significantly between groups over the 56 days, a slight reduction was observed in the HS3 group compared to other tested groups (HS0, HS1, HS6) after 28, 42, and 56 days. In accordance with our study, the survival rate of juvenile *P. dabryanus* (fed 0–2% lignite FA) or *O. mykiss* (fed 0–1.2% HA sodium salt) was not affected by the HS diet after 60 days, respectively [24,26]. Furthermore, Yamin et al. [27] did not observe any differences in survival rate or external abnormalities of *Carassius auratus* treated with various HS (leonardite, commercial HA sodium salt, RAS-derived) used in water (100 mg L^−1^, 100 mg L^−1^, 100% RAS water) and simultaneously in feed (20 g kg^−1^, 20 g kg^−1^, 100 g kg^−1^), after 45 days exposure, respectively. 

Nevertheless, it should be noted that indisputable positive effects of HS exposure on fish survival rate and fish performance were observed during various infection (parasitic, bacterial, virulent) challenges. For instance, oral administration of humus extract (0.2, 1, 5, 10, 20%) induced effective protection against *Aeromonas salmonicida* infection in *C. carpio* [53], as well as in the study of Abdel-Wahab et al. [21] who fed *C. carpio* with commercial HA inclusion (0.4, 0.8, 1%) and challenged them with *Aeromonas hydrophila.* Furthermore, [27] found that the addition of various HS (leonardite, commercial HA sodium salt, RAS-derived) into both water (20 mg L^−1^, 2 mg L^−1^, 100% RAS water, respectively) and feed (20 g kg^−1^, 20 g kg^−1^, 100 g kg^−1^) reduced *A. salmonicida* infection rates in *C. carpio*. Another study from [26] reported better resistance of *O. mykiss* against *Yersinia ruckeri* infection after 60 days of HA sodium salt feeding (0.3, 0.6, 1.2%), with the best results with 0.6% diet. In addition, Nakagawa et al. [30] observed effective protection against *Flavobacterium psychrophilum* infection in *Plecoglossus altivelis* fed by earth humus extract (0, 1, 5, 10%) for 30 days prior and 21 days during the bacterial infection.

Previous studies provided evidence that HS-rich feed or water additive may increase fish survival rate, for instance, due to fungistatic effects against *Saprolegnia parasitica* [18], or binding properties causing toxicity reduction in metals—cadmium [54] and copper [55], pollutants—ammonia and nitrite [56], or xenobiotics, and the associated damage of fish tissues. Furthermore, HS are known for their ability to stimulate the uptake of essential ions and decrease respiratory stress in acidic environments [14,57].

Furthermore, HS can be considered a possible mild chemical stressor—rather beneficial in mild concentrations [14]—as they had a quick recovery to HS-exposed *X. helleri* compared to the control group where fish growth stagnated after two weeks of stressful handling [25]. Lieke et al. [23] also confirmed this finding in their study with juvenile *O. mykiss* where the lowest stress impact on the course of plasma cortisol levels was observed in fish previously exposed to FA-rich water (50 mg C/L) for 28 days. According to Gao et al. [24], antioxidant activity—an extremely important defence mechanism in relation to stress living organism resistance—was elevated in juvenile *P. dabryanus* after use of lignite FA (0.5, 1.0, 1.5, 2%) feed for 60 days.

For these HS properties, some authors consider HS a suitable substance to replace antibiotics in aquaculture [26,58].

## 5. Conclusions 

Although our results provided no conclusive evidence that oral administration of Siberian leonardite HS improved growth performance and survival rate of *C. gariepinus*, moderately positive effects were observed regarding health status and good antioxidant parameters, especially in the HS3 group. With regard to other HS studies on fish, it can be summarized that different HS sources applied in both aquafeed (0.3–20%) and water (1.5–500 mg C/L) might have contradicting (positive, none, or negative) effects on fish growth and physiological or health conditions. It should be noted that the HS effect is probably not only HS-specific (level of substance, HS source, FA:HA ratio, chemical structure, application method, length of exposure) and animal-specific (species, age, health condition before exposure), but also farm-environment-specific (e.g., water quality, water change, water parameters, as the HS may bind/react with contained elements/compounds). Unfortunately, some fish studies do not clearly describe information on the HS source structure (i.e., HS content; HU, FA, or HA ratio; composition of major elements C, H, N, and S; molecular weight or water solubility). Further research of different HS applications on fish performance together with more information on approximate HS structure is required (for instance, to test short-term HS exposure in other fish species, to verify if long-term HS exposure has stronger impact, and to explain the existing HS-contradicting effects in fish, as well as the function of different HS sources or structures on fish performance).

## Figures and Tables

**Table 1:** Proximate elemental composition (%) of tested Siberian leonardite.

Concentration	Elements
25–40%	C, K
1–2%	H, Ca, Si, Fe
0.1–1%	N, Al, S, Cl, Sr
0.01–0.1%	Na, Mg, Ti, V, Cr, Ni, Zr, Sb, Cs
0.001–0.01%	Zn, Ga, Br, Rb, Y, Nb, Cd, In, Sn, Te, Hf, Hg
<0.001%	P, Mn, Co, Cu, Ge, As, Se, Mo, Ag, I, Ba, La, Ce, Ta, W, Tl, Pb, Bi, Th, U

**Table 2:** Proximate composition (%) of experimental diets HS0, HS1, HS3 and HS6.

Components	HS0	HS1	HS3	HS6
Dry matter (% g of feed)	90.39	90.43	90.52	90.64
Crude protein (% of feed)	42.00	42.00	42.01	42.02
Crude lipid (% of feed)	12.00	12.00	12.00	12.00
Ash (% of feed)	6.80	6.82	6.87	6.95

**Table 3:** Fish biomass in tank (B), individual body weight (BW), total body length (TL and SL), specific growth rate (SGR), absolute growth rate (AGR), food conversion ratio (FCR), daily feeding rate (DFR), coefficient of variation (CV), condition factor (K), thermal growth coefficient (TGC), somatic indices (HSI, SSI), and overall mortality (OM) of *C. gariepinus* juveniles after 0 and 56-days of feeding with four experimental HS diets (HS0, HS1, HS3, HS6).

Parameters	Initial	HS0	HS1	HS3	HS6	*F^test^*	*n*	*p*
Period (d)	Day 0	Day 56	Day 56	Day 56	Day 56	Day 56	Day 56	Day 56
B (kg)	2.94 ± 0.09	10.71 ± 0.51	10.47 ± 0.86	11.14 ± 0.51	10.81 ± 0.84	0.82 ^1WA^	20	0.50
BW (g)	32.38 ± 1.96	173.85 ± 6.16	169.42 ± 9.27	174.53 ± 2.00	176.36 ± 12.63	1.83^KW^	20	0.61
TL (mm)	162.45 ± 3.36	272.35 ±4.02	271.37 ± 6.95	275.05 ± 2.07	274.01 ± 7.17	0.45 ^1WA^	20	0.72
SL (mm)	144.31 ± 3.33	242.12 ± 4.26	238.43 ± 6.24	247.10 ± 1.84	246.22 ± 6.63	3.07 ^1WA^	20	0.06
SGR (% d^−1^)	-	2.52 ± 0.87	2.52 ± 0.59	2.40 ± 0.40	2.92 ± 0.70	0.57 ^1WA^	20	0.64
AGR (% d^−1^)	-	2.53 ± 0.13	2.46 ± 0.16	2.53 ± 0.03	2.56 ± 0.21	0.37 ^1WA^	20	0.77
FCR	-	1.11 ± 0.16	1.17 ± 0.30	1.06 ± 0.11	1.03 ± 0.14	3.72 ^KW^	20	0.29
DFR (feed fish^−1^day^−1^)	-	2.40 ± 0.07	2.35 ± 0.11	2.38 ± 0.06	2.42 ± 0.14	0.47 ^1WA^	20	0.71
CV (%)	25.60 ± 2.58	47.76 ± 4.62	42.60 ± 5.30	42.41 ± 3.00	40.55 ± 3.31	2.76 ^1WA^	20	0.08
K	0.75 ± 0.01	0.86 ± 0.02	0.85 ± 0.02	0.84 ± 0.01	0.86 ± 0.02	1.34 ^1WA^	20	0.30
TGC		1.59 ± 0.08	1.57 ± 0.08	1.58 ± 0.03	1.58 ± 0.07	0.06 ^1WA^	20	0.98
HSI (%)	-	1.33 ± 0.38	1.23 ± 0.04	1.28 ± 0.04	1.19 ± 0.04	0.34 ^1WA^	20	0.80
SSI (%)	-	0.04 ± 0.02	0.04 ± 0.02	0.04 ± 0.02	0.04 ± 0.01	0.56 ^1WA^	20	0.65
OM (%)	-	31.56 ± 1.49	31.33 ± 3.88	29.11 ± 2.77	31.11 ± 5.03	0.50 ^1WA^	20	0.68

Values are presented as mean ± SD. Number of considered experimental units (*n*). One-way ANOVA (^1WA^) or Kruskal-Wallis (^KW^) test results are power of test (*F*), and level of significance (*p*). Different superscripts (a, b) indicate significant differences (Tukey HSD test, *p* < 0.05) within one row.

**Table 4:** Biochemical parameters (total proteins, TP; alanine aminotransferase, ALT; aspartate aminotransferase, AST; cholesterol, CHOL; triglycerides, TAG; lactate dehydrogenase, LDH; glucose, GLU) and glutathione (reduced glutathione, GSH; oxidized glutathione, GSSG; glutathione ratio, GSH/GSSG) of *C. gariepinus* after 0 and 56 days of feeding with four experimental HS diets (HS0, HS1, HS3, HS6).

Parameters	Initial	HS0	HS1	HS3	HS6	*F*	*n*	*p*
Period (d)	Day 0	Day 56	Day 56	Day 56	Day 56	Day 56	Day 56	Day 56
TP (g L^−1^)	27.23 ± 4.95	30.14 ± 1.80	26.87± 8.88	32.29 ± 5.08	30.80 ± 4.43	1.90 ^KW^	40	0.59
ALT (ukat L^−1^)	3.36 ± 1.41	0.49 ± 0.27	0.35 ± 0.18	0.51 ± 0.20	0.53 ± 0.12	1.59 ^1WA^	40	0.21
AST (ukat L^−1^)	2.75 ± 0.82	2.26 ± 2.34	1.31 ± 0.46	1.40 ± 0.34	1.20 ± 0.29	3.54 ^KW^	40	0.32
CHOL (mmol L^−1^)	3.25 ± 0.65	2.93 ± 0.27	2.68 ± 0.34	2.82 ± 0.43	2.82 ± 0.51	0.62 ^1WA^	40	0.60
TAG (mmol L^−1^)	1.49 ± 1.30	1.34 ± 1.19	0.95 ± 0.22	1.71 ± 1.63	0.91 ± 0.32	3.13 ^KW^	40	0.37
LDH (ukat L^−1^)	6.03 ± 2.52	3.76 ± 1.76	2.73 ± 1.28	3.76 ± 1.64	2.69 ± 1.25	4.81 ^KW^	40	0.19
GLU (mmol L^−1^)	4.83 ± 3.85	4.73 ± 1.68^a^	4.78 ± 1.58^a^	4.59 ± 0.66^a^	3.20 ± 1.37^b^	2.99 ^1WA^	40	0.04
GSH (µM)	204.65 ± 41.28	187.67 ± 68.42	174.60 ± 51.89	170.78 ± 43.52	171.23 ± 29.77	0.25 ^1WA^	40	0.86
GSSG (µM)	43.89 ± 15.65	8.74 ± 4.20	8.18 ± 4.12	6.99 ± 4.00	8.61 ± 3.32	0.41 ^1WA^	40	0.74
GSH/GSSG (µM)	2.98 ± 1.26	24.90 ± 19.12	25.45 ± 16.14	30.90 ± 24.05	23.99 ± 19.02	1.39 ^KW^	40	0.71

Values are presented as mean ± SD. Number of considered experimental units (*n*). One-way ANOVA (^1WA^) or Kruskal-Wallis (^KW^) test results are power of test (*F*), and level of significance (*p*). Different superscripts (a, b) indicate significant differences (Tukey HSD test, *p* < 0.05) within one row.

## Data Availability

The data presented in this study is available on request from the corresponding author.

## Supplementary Materials

The following are available online at https://www.mdpi.com/article/10.3390/ani11082266/s1, Table S1: Fish biomass in tank (B), individual body weight (BW), total body length (TL and SL), coefficient of variation (CV), and condition factor (K) of *C. gariepinus* juveniles after 0 days of feeding with four experimental HS diets (HS0-HS6), Table S2: Fish biomass in tank (B), individual body weight (BW), total body length (TL and SL), specific growth rate (SGR), food conversion ratio (FCR), coefficient of variation (CV), condition factor (K), and overall mortality (OM) of *C. gariepinus* juveniles after 14 days of feeding with four experimental HS diets (HS0-HS6), Table S3: Fish biomass in tank (B), individual body weight (BW), total body length (TL and SL), specific growth rate (SGR), food conversion ratio (FCR), coefficient of variation (CV), condition factor (K), and overall mortality (OM) of *C. gariepinus* juveniles after 28 days of feeding with four experimental HS diets (HS0-HS6), Table S4: Fish biomass in tank (B), individual body weight (BW), total body length (TL and SL), specific growth rate (SGR), food conversion ratio (FCR), coefficient of variation (CV), condition factor (K), and overall mortality (OM) of *C. gariepinus* juveniles after 42 days of feeding with four experimental HS diets (HS0-HS6), Table S5: Biochemical parameters (total proteins, TP; alanine aminotransferase, ALT; aspartate aminotransferase, AST; cholesterol, CHOL; triglycerides, TAG; lactate dehydrogenase, LDH; glucose, GLU) and glutathione (reduced glutathione, GSH; oxidized glutathione, GSSG; glutathione ratio, GSH/GSSG) of *C. gariepinus* after 0 and 28 days of feeding with four experimental HS diets (HS0-HS6).
