# Supplementary material for: Effect of Humic Substances as Feed Additive on the Growth Performance, Antioxidant Status, and Health Condition of African Catfish (Clarias gariepinus, Burchell 1822)
Source: Animals (Basel). 2021 Jul 31;11(8):2266. doi: 10.3390/ani11082266 (PMC8388438; doi:10.3390/ani11082266)
Supplement: Supplementary file 1 [file animals-11-02266-s001.zip › animals-1274705-supplementary.pdf]

Supplementary data:

Supplement 1. Fish biomass in tank (B), individual body weight (BW), total body length (TL and SL), coefficient of variation (CV), and condition factor (K) of *C. gariepinus* juveniles after 0 days of feeding with four experimental HS diets (HS0-HS6).

**Parameters**	**HS0**	**HS1**	**HS3**	**HS6**	F^{test}	***n***	***P***
Period (d)	Day 0	Day 0	Day 0	Day 0	Day 0	Day 0	Day 0
B (kg)	2.88 ± 0.05	2.91 ± 0.14	2.98 ± 0.11	3.00 ± 0.05	1.92^{1WA}	20	0.17
BW (g)	32.12 ± 2.06	31.47 ± 3.01	32.83 ± 1.30	33.09 ± 1.47	0.62^{1WA}	20	0.61
TL (mm)	162.44 ± 3.61	161.23 ± 4.97	163.34 ± 2.61	162.79 ± 2.25	0.32^{1WA}	20	0.81
SL (mm)	143.99 ± 3.70	143.98 ± 4.75	144.71 ± 2.54	144.57 ± 2.33	0.06^{1WA}	20	0.98
CV (%)	26.06 ± 2.69	25.90 ± 2.82	24.70 ± 1.65	25.75 ± 3.18	0.27^{1WA}	20	0.85
K	0.75 ± 0.01	0.75 ± 0.00	0.75 ± 0.01	0.77 ± 0.02	2.63^{KW}	20	0.45

Values are presented as mean ± SD. Number of considered experimental units (*n*). One-way ANOVA (^{1WA}) or Kruskal-Wallis (^{KW}) test results are power of test (*F*), and level of significance (*P*). Different superscripts (a, b) indicate significant differences (Tukey HSD test, $P < 0.05$) within one row.

Supplement 2. Fish biomass in tank (B), individual body weight (BW), total body length (TL and SL), specific growth rate (SGR), food conversion ratio (FCR), coefficient of variation (CV), condition factor (K), and overall mortality (OM) of *C. gariepinus* juveniles after 14 days of feeding with four experimental HS diets (HS0-HS6).

**Parameters**	**HS0**	**HS1**	**HS3**	**HS6**	F^{test}	***n***	***P***
Period (d)	Day 14	Day 14	Day 14	Day 14	Day 14	Day 14	Day 14
B (kg)	4.30 ± 0.19	4.16 ± 0.20	4.33 ± 0.16	4.25 ± 0.21	0.84^{1WA}	20	0.49
BW (g)	53.34 ± 4.81	51.16 ± 2.30	50.45 ± 4.32	49.76 ± 3.82	0.78^{1WA}	20	0.52
TL (mm)	186.61 ± 5.52	184.15 ± 3.88	185.77 ± 4.43	183.38 ± 5.80	0.44^{1WA}	20	0.73
SL (mm)	168.02 ± 5.53	165.29 ± 3.92	167.44 ± 4.05	165.28 ± 5.38	0.45^{1WA}	20	0.72
SGR (% d^{-1})	3.61 ± 0.52	3.49 ± 0.52	3.05 ± 0.65	2.90 ± 0.58	1.79^{1WA}	20	0.19
FCR	0.98 ± 0.13	1.13 ± 0.09	1.06 ± 0.06	1.19 ± 0.27	5.13^{KW}	20	0.16
CV (%)	36.69 ± 3.18	35.18 ± 5.39	35.19 ± 8.01	39.99 ± 8.86	0.56^{1WA}	20	0.64
K	0.82 ± 0.01	0.82 ± 0.03	0.79 ± 0.02	0.81 ± 0.02	2.82^{1WA}	20	0.07
OM (%)	9.33 ± 1.49	8.89 ± 2.48	7.56 ± 3.28	5.56 ± 3.77	1.73^{1WA}	20	0.20

Values are presented as mean ± SD. Number of considered experimental units (*n*). One-way ANOVA (^{1WA}) or Kruskal-Wallis (^{KW}) test results are power of test (*F*), and level of significance (*P*). Different superscripts (a, b) indicate significant differences (Tukey HSD test, $P < 0.05$) within one row.

Supplement 3. Fish biomass in tank (B), individual body weight (BW), total body length (TL and SL), specific growth rate (SGR), food conversion ratio (FCR), coefficient of variation (CV), condition factor (K), and overall mortality (OM) of *C. gariepinus* juveniles after 28 days of feeding with four experimental HS diets (HS0-HS6).

Parameters	HS0	HS1	HS3	HS6	F^{test}	n	P
Period (d)	Day 28	Day 28	Day 28	Day 28	Day 28	Day 28	Day 28
B (kg)	6.06 ± 0.15	5.88 ± 0.35	6.23 ± 0.21	6.21 ± 0.28	1.96^{1WA}	20	0.16
BW (g)	83.86 ± 3.63	78.75 ± 8.04	77.68 ± 8.52	75.65 ± 2.96	1.54^{1WA}	20	0.24
TL (mm)	215.58 ± 4.42	209.94 ± 8.09	211.47 ± 6.98	209.76 ± 3.97	1.19^{1WA}	20	0.35
SL (mm)	192.77 ± 3.46	186.58 ± 7.62	185.11 ± 6.37	184.23 ± 3.84	2.67^{1WA}	20	0.08
SGR (% d^{-1})	3.25 ± 0.82	3.06 ± 0.75	3.07 ± 0.80	3.00 ± 0.61	0.10^{1WA}	20	0.96
FCR	1.09 ± 0.17	1.07 ± 0.09	1.01 ± 0.04	0.96 ± 0.09	1.36^{1WA}	20	0.29
CV (%)	37.30 ± 2.74	41.65 ± 5.82	45.80 ± 4.52	40.80 ± 5.59	2.62^{1WA}	20	0.09
K	0.84 ± 0.02	0.85 ± 0.02	0.82 ± 0.04	0.82 ± 0.02	1.60^{1WA}	20	0.23
OM (%)	14.67 ± 1.45	13.78 ± 2.30	12.44 ± 1.83	12.67 ± 2.68	1.73^{1WA}	20	0.20

Values are presented as mean ± SD. Number of considered experimental units (*n*). One-way ANOVA (^{1WA}) or Kruskal-Wallis (^{KW}) test results are power of test (*F*), and level of significance (*P*). Different superscripts (a, b) indicate significant differences (Tukey HSD test, $P < 0.05$) within one row.

Supplement 4. Fish biomass in tank (B), individual body weight (BW), total body length (TL and SL), specific growth rate (SGR), food conversion ratio (FCR), coefficient of variation (CV), condition factor (K), and overall mortality (OM) of *C. gariepinus* juveniles after 42 days of feeding with four experimental HS diets (HS0-HS6).

Parameters	HS0	HS1	HS3	HS6	F^{test}	n	P
Period (d)	Day 42	Day 42	Day 42	Day 42	Day 42	Day 42	Day 42
B (kg)	8.28 ± 0.29	8.15 ± 0.60	8.55 ± 0.28	8.29 ± 0.46	0.74^{1WA}	20	0.54
BW (g)	122.59 ± 12.24	119.19 ± 10.32	124.87 ± 8.08	117.94 ± 17.16	0.33^{1WA}	20	0.81
TL (mm)	242.65 ± 6.24	239.48 ± 10.25	246.09 ± 6.75	240.30 ± 10.51	0.58^{1WA}	20	0.63
SL (mm)	219.42 ± 5.55	216.17 ± 9.90	220.85 ± 6.31	216.10 ± 9.29	0.45^{1WA}	20	0.72
SGR (% d^{-1})	2.69 ± 0.48	2.97 ± 0.75	3.41 ± 0.64	3.12 ± 0.84	0.96^{1WA}	20	0.43
FCR	1.07 ± 0.09	1.02 ± 0.11	1.05 ± 0.04	1.15 ± 0.10	2.13^{1WA}	20	0.14
CV (%)	44.35 ± 8.02	43.00 ± 8.45	36.55 ± 3.70	39.91 ± 2.95	1.53^{1WA}	20	0.25
K	0.86 ± 0.05	0.87 ± 0.04	0.84 ± 0.02	0.85 ± 0.02	1.17^{1WA}	20	0.35
OM (%)	24.22 ± 2.41	21.20 ± 1.48	18.20 ± 1.30	20.40 ± 2.79	3.02^{1WA}	20	0.06

Values are presented as mean ± SD. Number of considered experimental units (*n*). One-way ANOVA (^{1WA}) or Kruskal-Wallis (^{KW}) test results are power of test (*F*), and level of significance (*P*). Different superscripts (a, b) indicate significant differences (Tukey HSD test, $P < 0.05$) within one row.

Supplement 5. Biochemical parameters (total proteins, TP; alanine aminotransferase, ALT; aspartate aminotransferase, AST; cholesterol, CHOL; triglycerides, TAG; lactate dehydrogenase, LDH; glucose, GLU) and glutathione (reduced glutathione, GSH; oxidized glutathione, GSSG; glutathione ratio, GSH/GSSG) of *C. gariepinus* after 0 and 28 days of feeding reduced glutathione, with four experimental HS diets (HS0, HS1, HS3, HS6).

**Parameters**	**Initial**	**HS0**	**HS1**	**HS3**	**HS6**	***F***	***n***	***P***
Period (d)	Day 0	Day 28	Day 28	Day 28	Day 28	Day 28	Day 28	Day 28
TP (g L^{-1})	27.23 ± 4.95	35.81 ± 9.35	32.46 ± 2.14	33.52 ± 5.77	33.36 ± 6.54	0.32^{KW}	40	0.96
ALT (ukat L^{-1})	3.36 ± 1.41	0.99 ± 0.97	0.75 ± 0.14	0.87 ± 0.33	0.73 ± 0.24	1.83^{KW}	40	0.61
AST (ukat L^{-1})	2.75 ± 0.82	1.82 ± 1.88	1.35 ± 0.41	2.07 ± 1.15	1.49 ± 0.59	4.51^{KW}	40	0.21
CHOL (mmol L^{-1})	3.25 ± 0.65	3.33 ± 0.61	3.31 ± 0.53	3.10 ± 0.36	3.12 ± 0.41	0.58^{KW}	40	0.90
TAG (mmol L^{-1})	1.49 ± 1.30	1.03 ± 0.80	0.81 ± 0.16	1.10 ± 1.01	0.70 ± 0.19	3.81^{KW}	40	0.28
LDH (ukat L^{-1})	6.03 ± 2.52	2.96 ± 1.47	3.72 ± 1.14	4.73 ± 2.09	3.58 ± 1.04	2.42^{KW}	40	0.08
GLU (mmol L^{-1})	4.83 ± 3.85	6.21 ± 3.01^{a}	3.88 ± 0.96^{b}	3.86 ± 1.33^{b}	3.9 ± 1.34^{b}	3.89^{1WA}	40	0.02
GSH (μM)	204.65 ± 41.28	153.45 ± 65.75	133.97 ± 20.89	135.60 ± 29.1 2	133.62 ± 33.5	0.30^{KW}	40	0.96
GSSG (μM)	43.89 ± 15.65	13.63 ± 5.47^{a}	17.37 ± 4.01^{b}	9.08 ± 5.18^{a}	12.14 ± 6.43^{a}	4.15^{1WA}	40	0.01
GSH/GSSG (μM)	2.98 ± 1.26	13.44 ± 18.66^{ab}	6.03 ± 1.09^{a}	19.35 ± 16.02^{b}	11.78 ± 8.49^{ab}	10.78^{KW}	40	0.01

Values are presented as mean ± SD. Number of considered experimental units (*n*). One-way ANOVA (^{1WA}) or Kruskal-Wallis (^{KW}) test results are power of test (*F*), and level of significance (*P*). Different superscripts (a, b) indicate significant differences (Tukey HSD test, $P < 0.05$) within one row.
